# Tonguedness in speech: Lateral bias in lingual bracing

**DOI:** 10.1121/10.0024756

**Published:** 2024-02-06

**Authors:** Yadong Liu, Jahurul Islam, Kate Radford, Oksana Tkachman, Bryan Gick

**Affiliations:** 1 Department of Linguistics, University of British Columbia, Vancouver, British Columbia, V6T 1Z4, Canada; 2 California Institute of Technology, Pasadena, California 91125, USA; 3 Haskins Laboratories, New Haven, Connecticut 06511, USA yadong@alumni.ubc.ca, jahurul.islam@ubc.ca, kradford@caltech.edu, o.tkachman@alumni.ubc.ca, gick@mail.ubc.ca

## Abstract

This study examines the lateral biases in tongue movements during speech production. It builds on previous research on asymmetry in various aspects of human biology and behavior, focusing on the tongue's asymmetric behavior during speech. The findings reveal that speakers have a pronounced preference toward one side of the tongue during lateral releases with a majority displaying the left-side bias. This lateral bias in tongue speech movements is referred to as tonguedness. This research contributes to our understanding of the articulatory mechanisms involved in tongue movements and underscores the importance of considering lateral biases in speech production research.

## 1. Introduction

Many parts of the human body, including the hands, feet, eyes, and ears, are laterally symmetrical and laterally biased; that is, humans show preference for one of the paired body parts (Marcori *et al.*, 2019; Rogers and Andrew, 2002). This lateral bias is believed to optimize behavior and function of the body parts in question. For example, laterally biased eyes enable depth perception rather than double vision (McBeath and Sugar, 2005). However, it is possible for non-paired body parts to show a lateral bias as well (Benson and Laskin, 2001). In this study, we examined the lateral biases in the tongue movements during speech. The tongue is a muscular structure that is grossly symmetrical (Sanders and Mu, 2013). During speech, the tongue is held bilaterally against the molars and for some sounds, the hard palate (Gibbon *et al.*, 2010; Gick *et al.*, 2017; Lee *et al.*, 2015; Narayanan *et al.*, 1997). This bracing assists in certain tongue movements (Stone, 1991) and is generally maintained throughout running speech but released during some laterals and occasional low vowels (Gick *et al.*, 2017). This pattern has been observed across diverse languages and language families (Liu *et al.*, 2023). Some evidence suggests that the movements into and out of the braced posture are asymmetrical with the lowering of one side of the tongue leading, and the leading side is consistent within speakers (Gick *et al.*, 2017). This study tests whether there exists evidence for a strong lateral bias in tongue movements during speech and discusses the implications of such a bias.

## 2. Background

Asymmetry is a common feature of human biology and behavior that has been studied across a variety of domains. For example, McBeath and Sugar (2005) argue that symmetry fares better in evolution than asymmetry resulting from universal laws of balance. Selection for asymmetric traits, however, operates at multiple levels, where some are related to more universal aspects of the environment or genetics and others are learned.

One area of human asymmetry that has been extensively studied is cerebral lateralization, particularly, with respect to speech. For instance, Strauss and Wada (1983) conducted sodium amytal speech testing on patients with various handedness and footedness preferences. They found that the majority of patients with right-handed preferences had speech represented in the left hemisphere. For example, 82% of patients with damage to the left hemisphere had speech mediated with the left hemisphere. Right hemisphere dominance in speech production is rare and associated with larger alterations in cortical organization (Labache *et al.*, 2023). This lateral bias was found to persist even on damage to the left hemisphere in 82% of patients (Strauss and Wada, 1983). On the other hand, patients with left hemisphere damage and a history of left-handed motor/sensory preferences had right hemisphere dominance. The authors suggest that handedness and footedness are relevant predictors of cerebral speech dominance. Another aspect of human asymmetry is facial asymmetry. Borod and Caron (1980) investigated whether facedness could be related to motor control in the contralateral hemisphere or emotional expression centers linked to the right cerebral hemisphere. They recorded facial expressions of 51 adults and determined direction and degree of facial asymmetry by ratings on the 15-point scale of the relative intensity of the facial expression on each side of the face. They found significantly more individuals with left-sided asymmetry, indicating right cerebral dominance. There were no significant differences in facedness based on sex, handedness, or facial expression of emotion.

Recent research highlights the existence of constraints that contribute to the graded asymmetry of cerebral lateralization. For instance, Behrmann and Plaut (2015) summarize empirical evidence from diverse studies involving various populations, including children, adolescents, adults, left-handers, and individuals with developmental dyslexia. These studies, employing behavioral assessments, evoked response potential measurements, and functional imaging techniques, collectively support the notion that hemispheric lateralization is not a binary phenomenon but rather exhibits a graded organization, which emerges dynamically throughout the developmental process.

Asymmetry in tongue movements (tonguedness as mentioned in Block *et al.*, 1957) has received much less attention compared to asymmetry in paired body parts, but the evidence available to date is suggestive of asymmetry as well. Reiss and Reiss (2002) investigated lateral tongue movements in cats and found more movements to the right side of the tongue and concluded overall movement dominance to the right side. Recently, tonguedness has been observed in human speech. For example, Lee *et al.* (2015) examined the asymmetry of tongue bracing during the onset phase of alveolar stops using custom electropalatography (EPG) plates created for each participant. They found that lateral contact increased before alveolar stops and the lateral margins of the tongue provided an anchor point, giving stability during stop production and assisting in deceleration of the tongue during ballistic stop movement. The authors note that some people with speech disorders are not able to brace their tongues laterally to create accurate stop closures.

Gick *et al.* (2017) studied the asymmetry of tongue bracing in running speech, specifically the tendency for speakers to release contact on one side of the mouth versus the other side. Even though previous research only described tongue bracing in terms of contact and not active maintenance of tongue position, the authors found evidence for an active effort to ensure bracing is always in place. For example, coronal medial bracing is always in place before lateral bracing is released. Asymmetries were observed on the side on which contact is lost (in unilateral contact loss) and in the sequential order of contact loss (in bilateral contact loss). The authors argue that speakers actively brace their tongues to facilitate the mechanics of tongue movements, provide somatosensory feedback for tongue position, and form the aeroacoustic tube, which separates the central oral tract from the lateral buccal cavities.

In summary, existing research provides abundant evidence for asymmetry in various aspects of human biology and behavior. Investigations into cerebral lateralization, facial asymmetry, and tongue bracing demonstrate the intricate relationships between anatomy, motor control, and speech production. This research underscores the significance of recognizing asymmetry as a fundamental characteristic of human communication, offering insights into the underlying mechanisms that shape our speech production. The present study aims to bridge the gap in our existing knowledge of lateral bias in human bodies, even in non-paired organs like the tongue, by studying lateral tongue release in speech production, further expanding our understanding of the extent and implications of asymmetry in this domain.

## 3. Method

### 3.1 Materials and procedures

Thirty-eight native English speakers with no reported speech or hearing impairment participated in the study. The data were recorded with a video camera (Sony Cyber-shot DSC-RX100, Minato-Ku, Tokyo, Japan), supported by an instrument arm attached to the participant's chair. Participants were asked to read aloud a 1-min-long English passage while biting on a 10 mm bite block, made of stacked narrow wooden tongue depressors, on each side of their mouth. The passage, originally used by Liu *et al.* (2022), was specifically designed to avoid labial sounds, thereby preventing lip closure from obstructing the camera's view. The data collection methods largely followed the methods described by Liu *et al.* (2022).

**Fig. 1.**
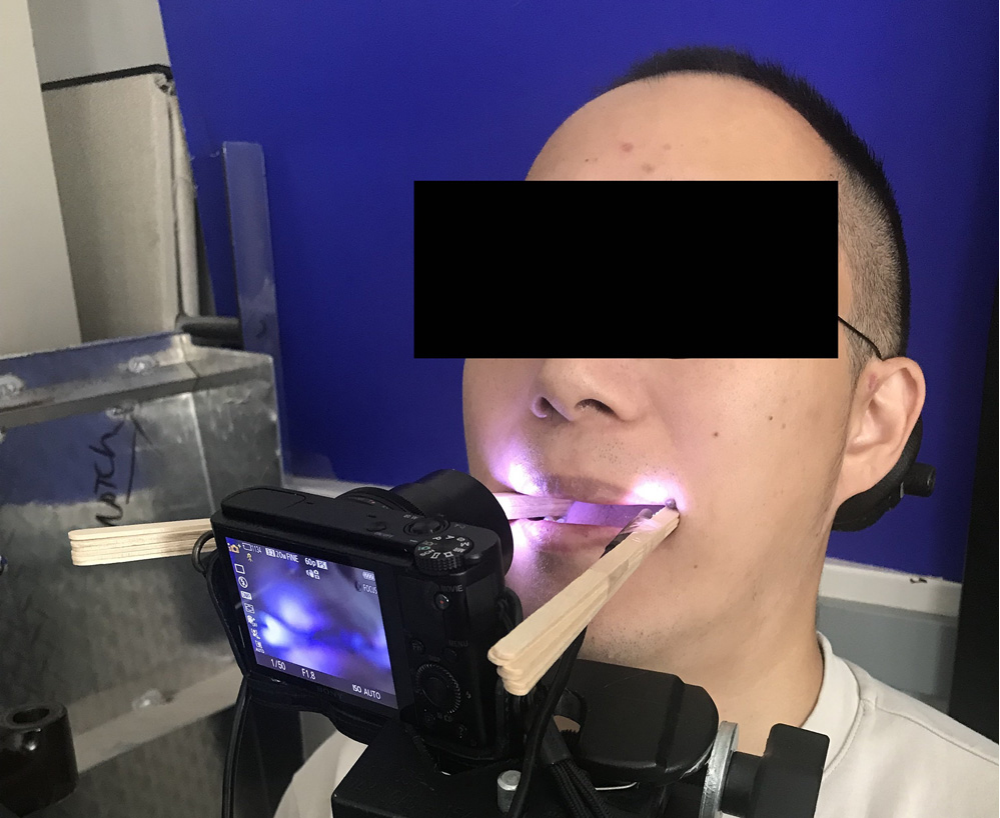
Experimental setup showing participant seated and fitted with bilateral bite block.

The bite blocks were used to keep the participants' mouths open throughout the reading session, allowing for clear visualization of tongue movements. To enhance visibility, a light-emitting diode (LED) was attached to each bite block, illuminating the inside of the oral cavity. During the recording session, participants were comfortably seated with their heads stabilized against a headrest. The bite blocks were positioned between the top and bottom molars on both sides of the mouth. To establish a baseline resting position for each participant's tongue, the video recording began a few seconds prior to the actual reading of the passage. Figure 1 provides an example of the experimental setup.

### 3.2 Analysis

**Fig. 2.**
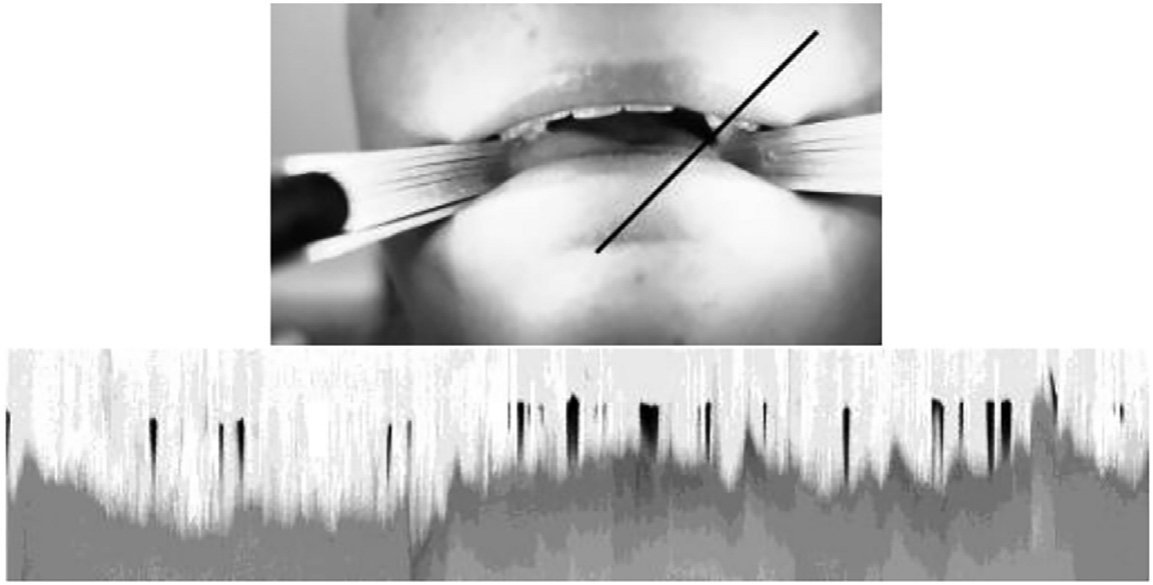
A frame of the video (top), where the black line indicates the location and angle of the slice used to produce the videokymograph (bottom) for measuring the release at the left side of the speaker. Each column of pixels corresponds to one frame of the video.

The video recordings were first converted into image sequences, which were then stacked and analyzed from different perspectives, producing videokymographs for the center, left, and right sides of the tongue using ImageJ (Schneider *et al.*, 2012; refer to Fig. 2). Each videokymograph represents the activity observed in a specific slice of the video over time, including interspeech pauses such as breathing and swallowing.

To facilitate analysis, the videokymograph images were converted into black-and-white format. In this representation, white areas correspond to the tongue and teeth while black areas correspond to an open oral cavity, indicating the absence of contact between the tongue and teeth. Subsequently, each videokymograph was cropped to include only the active speech portion.

To quantify the movement of the tongue away from the braced posture, a PYTHON script was employed. The script counted the number of black pixels in each column of the kymograph, which represented the distance of the tongue release from the braced position within one frame of the original video. This movement, referred to as release (Gick *et al.*, 2017), encompasses the sides of the tongue moving away from the braced posture. The black regions indicate the absence of contact between one side of the tongue and the upper teeth. The vertical extent of the black region reflects the distance between the side of the tongue and the teeth, whereas the width of the black region corresponds to the duration of the release. Consequently, a larger black region illustrates a larger movement away from the braced posture.

To investigate the potential relationship between handedness and tonguedness, we assessed participants' handedness preferences using the short form of the Edinburgh Handedness Inventory (Veale, 2014) and subsequently analyzed the correlation between handedness and tonguedness in our data.

## 4. Results

### 4.1 Lateral bias

**Fig. 3.**
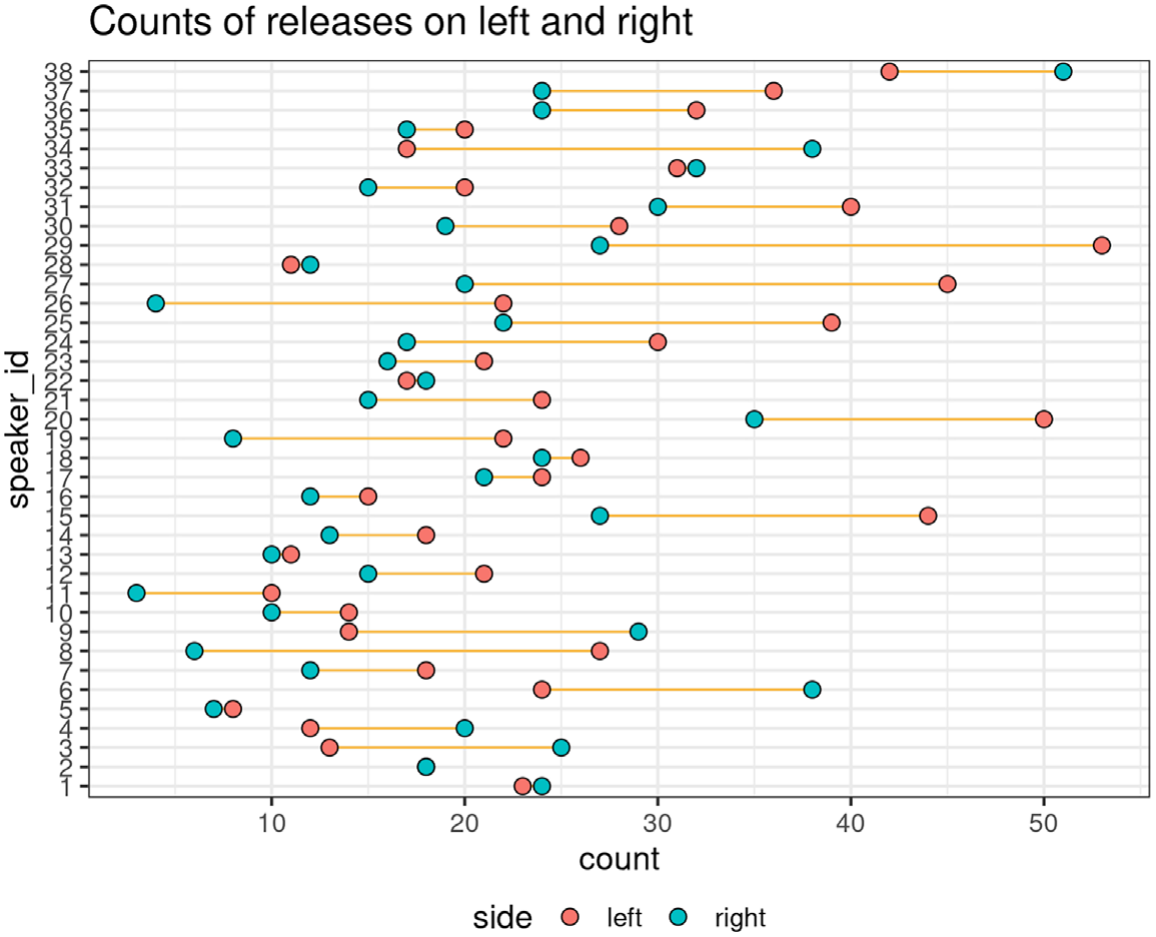
Raw counts of lateral releases on left and right sides for all speakers.

Figure 3 presents the number of black regions as raw counts of lateral releases on each side of the tongue (left and right) for all speakers in the study. The *x* axis shows how many times a speaker exhibited a release on either side of the tongue during the whole utterance, and the *y* axis displays individual speakers. Data from the left and right sides are differentiated with colors. As Fig. 3 shows, the majority of the speakers were noticeably asymmetric in terms of lateral release. That is, for most speakers, lateral releases did not always involve the movement of both sides of the tongue; rather, one side of the tongue was more frequently involved in lateral releases. A pairwise *t*-test (two-tailed, *N* = 38) was performed to assess the statistical significance of this asymmetry in speakers' preferences. There was a significant difference [*t*(2.76) = 37, *p* = 0.009] between the left and right sides of the tongue in terms of the counts of lateral releases.

Figure 3 also shows that there were more releases on the left side of the tongue, indicating a bias toward the left side of the tongue for most speakers. To compare results between speakers, we normalized the values in the following way: for each speaker, we took the difference in counts by subtracting the counts for the “right” from the “left” and then dividing the result by the total counts from right and left. This gives us a single metric for each speaker; we refer to this metric as measure of bias (MOB). A positive MOB indicates that the speaker has a preference for left-side releases (more releases on the left side than the right side) while a negative value indicates a right-side preference.

**Fig. 4.**
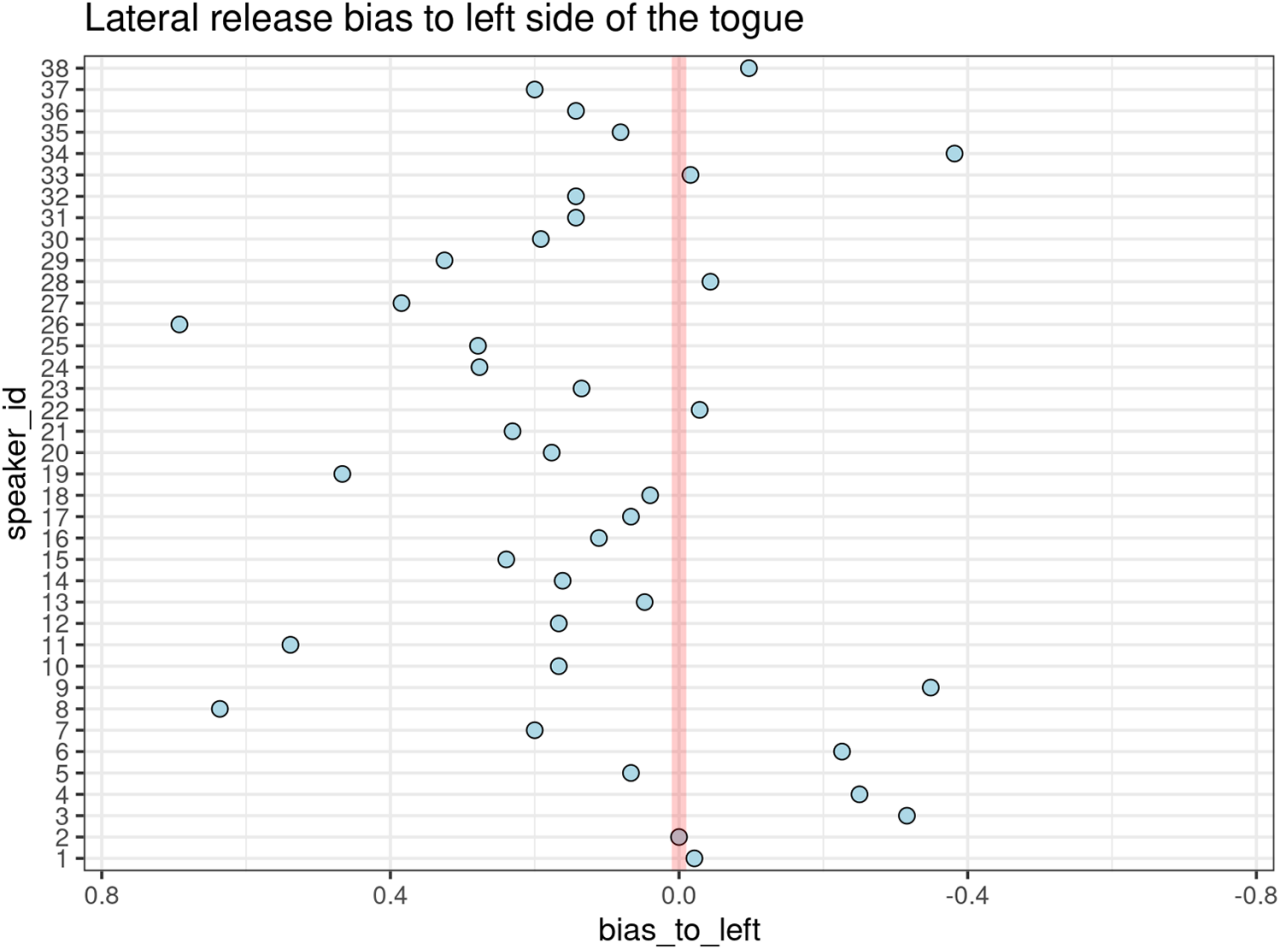
MOB in lateral tongue release (positive = left-biased and negative = right-biased).

Figure 4 presents the MOB values for all speakers in the study and shows most speakers (27 speakers) had a strong bias toward the left side (i.e., most speakers have positive MOB values) while only a few (8 speakers) had a strong bias toward the right side; 3–4 speakers (with MOB values close to zero) can be categorized as balanced speakers who release both sides of the tongue for lateral releases.

### 4.2 Tongue side dominance and handedness

The lateral biases in tongue movements observed in this study raise the question of tonguedness, that is, which side of the tongue is the “dominant” side. It is instructive to consider this question in the light of another prominent functional asymmetry in humans, handedness. Approximately 90% of the population exhibit a pronounced right-hand preference (Warren, 1980), relegating the left hand primarily to bimanual tasks (Takagi *et al.*, 2020). The handedness research has been marked by divergent perspectives on hand dominance in bimanual tasks. Although some studies propose that the nondominant hand excels in postural maintenance (Woytowicz *et al.*, 2018), others, notably Takagi *et al.* (2020), posit that the dominant limb exerts greater force to stabilize posture during bimanual tasks. In the context of the tongue, adhering to the former perspective would designate the side maintaining more lateral bracing and fewer releases as the nondominant side, whereas adopting the latter viewpoint would identify the side exhibiting stronger contraction as the dominant side. However, the hydrostatic properties of the tongue introduce a novel dynamic: when the dominant side contracts more forcefully, it can elevate the opposing side to a higher position, thereby enhancing its ability to maintain a bracing posture (see simulations in Azreen *et al.*, 2023). Consequently, both perspectives on hand dominance in bimanual tasks converge to suggest that the side of the tongue displaying greater bracing posture endurance should be classified as the nondominant side, whereas the side exhibiting a greater tendency to release should be designated as the dominant side.

We analyzed the correlation of participants' tonguedness and handedness. Given that all seven left-handed participants are also left-tongued, resulting in zero observations of right-tongued, left-handed participants, a Chi-square test is not applicable in this context. Consequently, we employed a Fisher exact test to examine the relationship between handedness and tonguedness, revealing a *p*-value of 0.0745. The results indicate that our study cohort does not exhibit a statistically significant correlation between handedness and tonguedness.

## 5. Conclusion

Previous literature shows the existence of asymmetry in lateral contact in typical (Gick *et al.*, 2017) and disordered speech (Gibbon and Wood, 2010) from individual speakers. The present study demonstrates that speakers exhibit a pronounced inclination toward one side when making lateral tongue releases in speech from a larger population. Only a minority of participants (4 out of 38) displayed a tendency to release both sides of their tongue during speech production. Participants consistently demonstrated lateral preferences in tongue positioning during releases. In particular, speakers exhibited a significant bias toward the left side of the tongue for lateral releases. Further, when speaking with a bite block, a condition imposed to maximize tongue movements by increasing the vertical distance between the tongue and palate/teeth, participants exhibited an increased frequency and magnitude of tongue release on one side of the tongue compared to the other side. This highlights the asymmetric nature of tongue movements during speech production.

In conclusion, our study explored one type of lateral bias in the human body, specifically in the tongue as a non-paired organ, by examining lateral tongue release during speech. This study sheds light on the strong lateral bias observed in speakers' tongue movements during speech and provides further evidence of lateral asymmetry of the human body. These findings contribute to our understanding of the articulatory mechanisms involved in lateral releases and underscore the importance of considering asymmetric tongue behavior in speech production research.

## Data Availability

The data that support the findings of this study are available from the corresponding author upon reasonable request.

## References

[1] Azreen, J. , Mayer, C. , Liu, Y. , Shamei, A. , Stavness, I. , and Gick, B. (2023). “ Biomechanical simulation of lateral asymmetry in tongue bracing,” J. Can. Acoust. Assoc. 51(3), 196–197.

[2] Behrmann, M. , and Plaut, D. C. (2015). “ A vision of graded hemispheric specialization,” Ann. N.Y. Acad. Sci. 1359(1), 30–46. 10.1111/nyas.12833 26199998 PMC13015954

[3] Benson, K. J. , and Laskin, D. M. (2001). “ Upper lip asymmetry in adults during smiling,” J. Oral Maxillofac. Surg. 59(4), 396–398. 10.1053/joms.2001.21874 11289169

[4] Block, I. , Disher, D. , and Froeschels, E. (1957). “ Tonguedness,” Folia Phoniatr. Logop. 9(1), 49–53. 10.1159/000262758 13427843

[5] Borod, J. C. , and Caron, H. S. (1980). “ Facedness and emotion related to lateral dominance, sex and expression type,” Neuropsychologia 18(2), 237–242. 10.1016/0028-3932(80)90070-6 7383316

[6] Gibbon, F. E. , Lee, A. , and Yuen, I. (2010). “ Tongue-palate contact during selected vowels in normal speech,” Cleft Palate-Craniofacial J. 47(4), 405–412. 10.1597/09-067.1 20590462

[7] Gibbon, F. E. , and Wood, S. E. (2010). “ Visual feedback therapy with electropalatography (EPG) for speech sound disorders in children,” in *Interventions in Speech Sound Disorders* ( Brookes, Baltimore, MD), pp. 509–536.

[8] Gick, B. , Allen, B. , Roewer-Després, F. , and Stavness, I. (2017). “ Speaking tongues are actively braced,” J. Speech. Lang. Hear. Res. 60(3), 494–506. 10.1044/2016_JSLHR-S-15-0141 28196377

[9] Labache, L. , Ge, T. , Yeo, B. T. , and Holmes, A. J. (2023). “ Language network lateralization is reflected throughout the macroscale functional organization of cortex,” Nat. Commun. 14(1), 3405. 10.1038/s41467-023-39131-y 37296118 PMC10256741

[10] Lee, A. , Gibbon, F. E. , and Oebels, J. (2015). “ Lateral bracing of the tongue during the onset phase of alveolar stops: An EPG study,” Clin. Linguist. Phonet. 29(3), 236–245. 10.3109/02699206.2014.991449 25495013

[11] Liu, Y. , Luo, S. , Łuszczuk, M. , Mayer, C. , Shamei, A. , de Boer, G. , and Gick, B. (2022). “ Robustness of lateral tongue bracing under bite block perturbation,” Phonetica 79(6), 523–549. 10.1515/phon-2022-0001 36974956 PMC10065199

[12] Liu, Y. , Tong, F. , de Boer, G. , and Gick, B. (2023). “ Lateral tongue bracing as a universal postural basis for speech,” J. Int. Phonet. Assoc. 53(3) 712–727. 10.1017/S0025100321000335

[13] Marcori, A. J. , Grosso, N. d. S. , Porto, A. B. , and Okazaki, V. H. A. (2019). “ Beyond handedness: Assessing younger adults and older people lateral preference in six laterality dimensions,” Laterality: Asymmetries Body, Brain Cognit. 24(2), 163–175. 10.1080/1357650X.2018.1495725 29975175

[14] McBeath, M. K. , and Sugar, T. G. (2005). “ Natural selection of asymmetric traits operates at multiple levels,” Behav. Brain Sci. 28(4), 605–606. 10.1017/S0140525X05390108

[15] Narayanan, S. S. , Alwan, A. A. , and Haker, K. (1997). “ Toward articulatory-acoustic models for liquid approximants based on MRI and EPG data. Part I. The laterals,” J. Acoust. Soc. Am. 101(2), 1064–1077. 10.1121/1.418030 9035398

[16] Reiss, M. , and Reiss, G. (2002). “ Tonguedness in cats,” Percept. Mot. Skills 94(1), 152–152. 10.2466/pms.2002.94.1.152 11883554

[17] Rogers, L. J. , and Andrew, R. (2002). *Comparative Vertebrate Lateralization* ( Cambridge University Press, Cambridge, UK).

[18] Sanders, I. , and Mu, L. (2013). “ A three-dimensional atlas of human tongue muscles,” Anat. Rec. 296(7), 1102–1114. 10.1002/ar.22711 PMC3687025 23650264

[19] Schneider, C. A. , Rasband, W. S. , and Eliceiri, K. W. (2012). “ NIH image to ImageJ: 25 years of image analysis,” Nat. Methods 9(7), 671–675. 10.1038/nmeth.2089 22930834 PMC5554542

[20] Stone, M. (1991). “ Toward a model of three-dimensional tongue movement,” J. Phonet. 19(3-4), 309–320. 10.1016/S0095-4470(19)30347-X

[21] Strauss, E. , and Wada, J. (1983). “ Lateral preferences and cerebral speech dominance,” Cortex 19(2), 165–177. 10.1016/S0010-9452(83)80012-4 6884038

[22] Takagi, A. , Maxwell, S. , Melendez-Calderon, A. , and Burdet, E. (2020). “ The dominant limb preferentially stabilizes posture in a bimanual task with physical coupling,” J. Neurophysiol. 123(6), 2154–2160. 10.1152/jn.00047.2020 32348682

[23] Veale, J. F. (2014). “ Edinburgh handedness inventory–short form: A revised version based on confirmatory factor analysis,” Laterality: Asymmetries Body, Brain Cognit. 19(2), 164–177. 10.1080/1357650X.2013.783045 23659650

[24] Warren, J. M. (1980). “ Handedness and laterality in humans and other animals,” Physiol. Psychol. 8(3), 351–359. 10.3758/BF03337470

[25] Woytowicz, E. J. , Westlake, K. P. , Whitall, J. , and Sainburg, R. L. (2018). “ Handedness results from complementary hemispheric dominance, not global hemispheric dominance: Evidence from mechanically coupled bilateral movements,” J. Neurophysiol. 120(2), 729–740. 10.1152/jn.00878.2017 29742023 PMC7132323

